# Probiotics: Potential Benefits and Safety in Hematological Malignancies

**DOI:** 10.31661/gmj.v13i.3149

**Published:** 2024-01-21

**Authors:** Mahdiyar Iravani Saadi, Mani Ramzi, Nastaran Fooladivanda, Sholeh Afshinpour, Zahra Ghahramani, Maryam Ahmadyan, Nadiya Kheradmand, Hourvash Haghighinejad, Zahra Rahimian

**Affiliations:** ^1^ Hematology Research Center, Shiraz University of Medical Sciences, Shiraz, Iran; ^2^ Hematology, Oncology and Bone Marrow Transplantation Department, Shiraz University of Medical Sciences, Shiraz, Iran; ^3^ Family Medicine Department, Shiraz University of Medical Sciences, Shiraz, Iran

**Keywords:** Probiotics, Hematologic Malignancy, Chemotherapy, Radiation Therapy, Hematopoietic Stem Cell Transplantation, HSCT, GVHD

## Abstract

Cancer remains one of the most significant global health challenges, with increasing incidence and mortality rates and a substantial socioeconomic burden. Traditional cancer treatments such as chemotherapeutic drugs and radiation therapies, while effective, have significant adverse effects on the body, prompting the search for less invasive treatment options. In recent years, probiotics have emerged as a promising alternative in the prevention and treatment of cancer and its associated complications, especially in gastrointestinal malignancies. Probiotics have been found to have several beneficial effects on the body, including the ability to mitigate the gastrointestinal side effects of cancer treatments by restoring gut microbiota balance and improving intestinal barrier function. By reducing inflammation and enhancing the immune response, probiotics can also help alleviate other cancer-related symptoms and improve the overall well-being of cancer patients. Despite their potential benefits, the efficacy and safety of probiotics in immunocompromised patients remain uncertain, and caution must be exercised during their administration. Further research is needed to determine the optimal clinical use, safety, and efficacy of probiotics in cancer treatment.

## Introduction

Cancer poses the highest clinical, social, and economic burden in terms of cause-specific Disability-Adjusted Life Years (DALYs) among all human diseases [1]. In 2020, there were an estimated 1.3 million new hematological cancers, with 700,000 deaths worldwide. The incidences of most hematological cancers increase with age [2][3]. Despite significant advancements in cancer treatment, chemotherapy and radiotherapy continue to cause substantial side effects [4][5], which can severely affect patients’ quality of life. One of the main concerns with chemotherapy is that it targets not only rapidly dividing cancer cells but also certain normal cells, such as intestinal epithelium [6], which can lead to various side effects. Similarly, radiation injury can lead to various pathologic lesions that can accumulate with time and manifest decades after exposure [7][8], including hematological complications, such as myelodysplasia and acute myeloid leukemia. Moreover, hematologic therapies, such as chemotherapy and immunosuppression, may induce or worsen dysbiosis, which can lead to disease progression and infectious complications [9][10][11][12].

The socioeconomic inequalities in cancer are also widening [13], with the burden of cancer falling more heavily on underserved populations, as they may not have access to the latest treatments or face challenges in accessing healthcare services. Therefore, developing less invasive ways to treat cancer is essential to improving survival rates and reducing the disparities in cancer outcomes.

One approach that has gained attention in recent years is the use of natural sources with anti-carcinogenic effects, such as probiotics [14][15][16]. Studies have investigated the potential of probiotics for the prevention and treatment of various human diseases [17]. Beneficial mechanisms identified include regulating intestinal flora, enhancing intestinal barrier function, protecting the intestinal epithelium from invasion by pathogens, and strengthening immune function [18][19].

While some experts consider probiotics an alternative treatment for various cancers, it’s important to note that cancer patients have compromised immunity caused by primary diseases, chemotherapy, and radiotherapy. As a result, the effects of probiotics may differ from those of healthy people, and several critical concerns need to be addressed [20]. Therefore, this study aims to evaluate the potential role of probiotics in the treatment of hematological cancer patients.

## Probiotics’ Roles

Humans coexist with a highly complex and diverse microbiome consisting of bacteria, fungi, and viruses that have coevolved with us over millions of years. This microbiome comprises approximately ten times more bacterial cells than human cells and contains over 100 times the genomic content of the human genome [21][22]. Dysbiosis, an imbalance in the gut microbiome, can lead to the activation of pro-inflammatory immune responses and trigger various disease processes, including cancer [23][24]. Several definitions for probiotics, prebiotics, and synbiotics have been proposed in scientific literature. However, the most accurate description defines them as beneficial microorganisms that inhabit the gut and provide internal nourishment to the host body, ultimately promoting a healthy gut microbiome and overall well-being [25][26].

Probiotics have been shown to modulate the gut microbiota and influence the host immune system through various pathways. One of these pathways is the production of short-chain fatty acids (SCFAs) by certain probiotic strains. SCFAs such as butyrate have been shown to promote the differentiation and function of regulatory T cells (Tregs) in the gut, leading to the suppression of inflammatory responses and the maintenance of immune homeostasis [27][28]. Another pathway through which probiotics can modulate the host immune system is by directly interacting with immune cells in the gut-associated lymphoid tissue (GALT)[29]. For instance, certain strains of lactobacilli have been shown to activate dendritic cells (DCs) in the GALT, leading to the production of pro-Th1/Th17 cytokines (TNFα, IL-1β, IL-12p70, IL-12p40, and IL-23), which in turn promote the differentiation and function of Tregs [30].

A key immune pathway of probiotic effects is through the regulation of inflammatory responses. Probiotics can modulate the production of inflammatory cytokines, such as interleukin-6 (IL-6) and tumor necrosis factor-alpha (TNF-alpha) [30][31]. This can lead to a reduction in chronic inflammation, which is associated with many chronic diseases, including inflammatory bowel disease and metabolic disorders [32].

**Figure-1:**
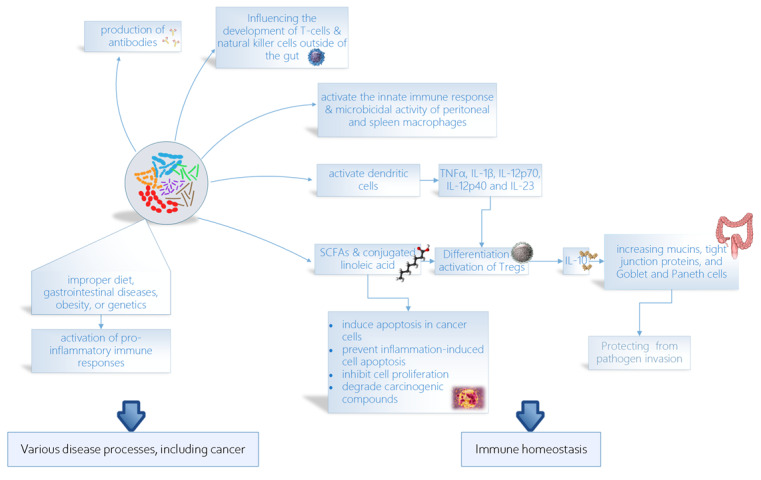
Figure 1. The figure highlights the key roles of probiotics in cancer development, immune homeostasis, and immune regulation. They can prevent cancer development by reducing inflammation and boosting the immune system. Probiotics can also help to maintain immune homeostasis and regulate the immune system, which may improve the response to cancer treatment.

Probiotics activate regulatory T cells that release IL-10 [33] and reinforce the intestinal barrier by increasing mucins, tight junction proteins, and Goblet and Paneth cells [34]. This can help to prevent the translocation of harmful pathogens into the bloodstream and reduce the risk of systemic infection[35]. Probiotics activate the innate immune response by interacting with IECs and macrophages and increasing the microbicidal activity of peritoneal and spleen macrophages [36][37]. In addition to their effects on the gut immune system, probiotics have also been shown to modulate systemic immune function by influencing the development and function of immune cells outside of the gut, such as T cells and natural killer cells [38]. Finally, probiotics can influence the production of antibodies, which are essential for fighting infections. Probiotics can stimulate the production of secretory IgA, which is the predominant immunoglobulin in the gut and plays a crucial role in protecting against pathogens [39] (Figure-1).

## Prevention

Intestinal dysbiosis, which can result from a variety of factors such as improper diet, gastrointestinal diseases, obesity, or genetics [40], has been associated with the development of both local gastrointestinal cancers and tumors in distant sites of the body [41]. Probiotics, however, have been found to have anticarcinogenic activity, which can be attributed to their ability to modify the composition of the intestinal microbiota, produce compounds with anticarcinogenic activity (such as SCFAs and conjugated linoleic acid), inhibit cell proliferation, induce apoptosis in cancer cells, influence other mutagenic and carcinogenic factors, bind and degrade carcinogenic compounds in the intestinal lumen, immunomodulate, improve the intestinal barrier [24], and prevent inflammation-induced cell apoptosis [42]. Some strains of probiotics can be used for cancer prevention or as an adjuvant treatment during anticancer chemotherapy by modulating the intestinal microbiota and immune response [24].

In animal studies, the treatment of Bacillus polyfermenticus, Bifidobacterium infantum, bifidum, and Lactobacillus acidophilus, casei, lactis, plantarum, rhamnosus, salivarius significantly inhibited the development of colon cancer in rats or mice injected with carcinogenic 1,2-dimethylhydrazine[24]. Kefir is recommended for people with adult lymphoblastic leukemia because of its pro-apoptotic properties [43]. Furthermore, in leukemia patients, kefir consumption inhibits cell proliferation and enhances apoptosis [44][45]. According to Chiu, Lactobacillus casei rhamnosus generated soluble bacterial components that were shown to increase apoptosis in a human monocytic leukemia cell line [46].

## Hematological Malignancies

The majority of these patients undergo chemotherapy or immunotherapy, and many receive antibiotic prophylaxis or other treatments that influence the composition and diversity of their gut microbiota. As a result, investigating the microbiome in hematological patients is particularly challenging.

Emerging data suggest that microbiota influence the development of multiple myeloma (MM)[47]. Superior treatment outcomes for MM correlate with a higher abundance of commensal microbiota capable of influencing inflammatory responses through the production of butyrate[48]. In patients with hematologic malignancies, higher levels of diversity of the gut microbiota correlate with superior outcomes after hematopoietic stem cell transplantation. Calcinotto and colleagues have shown the role of Prevotella heparinolytica, a commensal bacterium, in the development of multiple myeloma (MM) by inducing an eosinophil-mediated inflammatory response and promoting the development of Th17 cells that migrate to the bone marrow to support tumor growth [47].

In Hodgkin lymphoma (HL) patients, a decrease in gut microorganism diversity has been linked to a reduced Th1 response and an enhanced Th2 response [49][50]. Both an increase in Th2 cytokines and IgE and a reduction in cytotoxic T cells and NK cells in HL might indicate that these patients are unable to make the Th2-to-Th1 switch [51]. This shift in immune response may be due to a lack of early childhood fecal-oral exposures, leading to decreased exposure to infections during childhood. The decrease in gut microorganism diversity could also be due to the lymphoma itself or the therapies received by the patients, serving as an early risk factor [50].

Additionally, a study using probiotic fermentation technology found that kefir grain product (Lactobacillus) increased apoptosis in cancer cells in a dose-dependent manner, indicating a potential treatment approach for multidrug-resistant leukemia [52]. Another study showed that kefir consumption inhibited cell proliferation and enhanced apoptosis in leukemia patients [45]. Similarly, Lactobacillus casei rhamnosus was found to increase apoptosis in a human monocytic leukemia cell line [46]. Recent studies have suggested that the gut microbiome may play a role in the development and progression of MDS, a group of bone marrow disorders that can lead to leukemia. For instance, a study found that patients with MDS had alterations in their gut microbiome composition compared to healthy individuals, with a decrease in certain beneficial bacteria, such as Faecalibacterium prausnitzii and Roseburia spp. These bacteria are known to play a role in maintaining gut barrier integrity, regulating inflammation, and modulating the immune response. Moreover, the gut microbiome has been shown to affect the efficacy of chemotherapy in MDS patients, with certain bacteria making chemotherapy less effective.

Similarly, the gut microbiome has been linked to the development and progression of CLL. A study found that antibiotics that modulate intestinal microbiota affect the efficacy of antineoplastic treatment in patients with relapsed lymphoma treated with cisplatin and patients with CLL treated with cyclophosphamide[53].

## HSCT and GVHD

There is growing evidence that the commensal microbiome is frequently dysregulated following allo-SCT and that this dysbiosis can predispose to adverse clinical outcomes, especially acute intestinal GVHD and reduced overall survival [54]. Human trials and animal studies have proven that a decrease in intestinal bacterial diversity is associated with the occurrence of GVHD[55]. Metabolites produced by intestinal bacteria, such as lipopolysaccharides, SCFAs, and secondary bile acids, can affect the development of GVHD through direct or indirect interactions with immune cells. The targeted damage of GVHD on intestinal stem cells and Paneth cells results in intestinal dysbiosis or dysbacteriosis [56].

Solid organ transplantation is associated with a high risk of infections, which can be life-threatening. Probiotics have been found to modulate the immune response and enhance the body’s natural defense against infections. Studies have suggested that probiotics may reduce the incidence of infections after solid organ transplantation and improve overall outcomes [57]. Gut cleansing treatment was found to reduce the incidence of acute gastrointestinal GvHD in children having allo-HCT in a multicenter research [58]. A significant decrease of commensal anaerobes (primarily Faecalibacterium prausnitzii—beneficial, butyrate-producing bacterium) and an increase of opportunistic bacteria have been found in children with aGvHD [59][60][61]. Furthermore, Biagi et al. found that young patients with gut aGvHD had altered gut flora [62].

Although more research is warranted, beneficial associations of Lactobacillales, Clostridiales, the Eubacterium limosum group and genus Blautia, Bacteroidetes species, and Clostridium clusters IV and XIVa have been observed in multiple studies. In contrast, enterococcal spp., proteobacterial spp., and C difficile have been associated with worsened GVHD outcomes [54]. Oral administration of Lactobacillus rhamnosus GG in drinking water before and after hematopoietic stem cell transplantation resulted in reduced bacterial translocation, improved survival, and reduced acute GVHD pathogenesis in murine models. Administration of butyrate-producing Clostridia spp. strains before and after allogeneic hematopoietic stem cell transplantation with an MHC mismatched model demonstrated a significant increase of butyrate in the intestine and a better survival rate [63].

## Chemo and Radiotherapy

Chemotherapy and radiotherapy have been shown to alter the gut microbiota, leading to diarrhea, nausea, and vomiting. Probiotics, particularly Lactobacillus and Bifidobacterium species, have been found to mitigate these side effects by restoring the gut microbiota’s balance. Studies have demonstrated that probiotics can reduce the severity and frequency of chemotherapy-induced diarrhea, improve intestinal permeability, and enhance immune function. Probiotics have been found to improve the integrity of the gut barrier, reduce inflammation, and promote healing of the intestinal mucosa. Studies have shown that probiotics can alleviate symptoms of radiation-induced enteritis, such as diarrhea, abdominal pain, and bloating [64]. Intestinal radiosensitivity is significantly linked to the gut microbiota [65]. Specific bacterial taxa also have been shown to contribute to GI tract recovery after radiation. Lachnospiraceae and Enterococcaceae are essential to maintain GI tract integrity and facilitate immune reconstitution long-term after radiation exposure [63].

Cancer patients are at increased risk of recurrent Clostridioides difficile infection (rCDI) due to malignancy itself, cancer therapy, and frequent antibiotic use and have a lower response rate to standard oral antibiotics. Fecal microbiota transplantation (FMT) is safe, well-tolerated, and efficacious in treating rCDI in selected cancer patients. However, additional antibiotic use for complications from chemotherapy or immunosuppression negatively affects the efficacy of FMT[66]

## Concerns

Nevertheless, the efficacy of probiotics in immunocompromised patients is still questionable. Using probiotics-enriched yogurt in a case report of a patient with autologous hematopoietic stem cell transplantation for treating acute promyelocytic leukemia resulted in unexpected Lactobacillus rhamnosus GG sepsis [67]. It is important to note that probiotics should be used cautiously in patients with compromised immune systems, such as those undergoing chemotherapy or radiotherapy. In one case, a two-year-old boy with neutropenia and increased inflammation after being diagnosed with acute B-cell lymphoblastic leukemia was found to have Bifidobacterium breve, which was not found in the child’s diet and could have led to serious infections [68]. In line with that, another clinical study reported bloodstream infections with Lactobacillus bacteremia in patients with autologous and allogeneic HSCT within the first 100 days post-HSCT, implying the toxicities from immunosuppression by conditioning regimens and mucosal disruption could contribute to bacteremia from probiotics consumption [63]. A study of children undergoing bone marrow transplantation found Lactobacillus bacteremia attributed to probiotic use [69]. Three probiotics (Lacticaseibacillus rhamnosus GG, Lactiplantibacillus plantarum, and Lacticaseibacillus paracasei) were directly linked with blood isolates from bacteremia patients using molecular identification assays [70]. Another study reported recurrent Lactobacillus bacteremia causing multiple episodes of fever of unknown origin in a patient with leukemia [71].

In a mouse model, Prevotella heparinolytica, a commensal bacterium, enhanced the development of Th17 cells invading the gut. These cells then moved to the bone marrow, where they supported tumor growth. Prevotella heparinolytica has also been shown to enhance the development of MM by inducing an eosinophil-mediated inflammatory response [47].

In a systematic review of Infectious complications following probiotic ingestion, the genus Saccharomyces was the most frequent, followed by Lactobacillus, Bifidobacterium, Bacillus, Pedioccocus and Escherichi [72].

In addition to the risks associated with probiotic use in immunocompromised patients, there is also growing concern about the potential impact of the gut microbiome on cancer development and progression. studies have suggested that certain types of bacteria, including P. intermedia and F. nucleatum affect malignant transformation of colorectal adenomas and enhance migration and invasion of these cancer cells [73].

These examples underscore the importance of carefully considering the risks and benefits of probiotics in cancer patients, especially those who are immunocompromised due to chemotherapy or other treatments. While probiotics may have potential benefits for some patients, their use can also lead to serious infections and other adverse effects, particularly in vulnerable populations. It is therefore important for healthcare providers to be aware of these risks and to carefully evaluate the appropriateness of probiotics in each individual patient. While the precise mechanisms by which the gut microbiome influences cancer development and progression are still being explored, these findings highlight the importance of considering the potential risks and benefits of probiotic use in the context of cancer treatment. Further research can help better understand the complex interactions between the gut microbiome and the immune system, and to develop safe and effective strategies for modulating the microbiome in the context of cancer therapy. It can also elucidate the potential of microbiome-based interventions as adjuvant therapies to enhance the efficacy of standard cancer treatments. Additionally, exploring the long-term effects of microbiome modulation on cancer survivors’ health and overall well-being can provide valuable insights for survivorship care. By prioritizing well-designed clinical trials in this area, we can advance our knowledge and eventually translate microbiome research into improved outcomes for cancer patients.

The heterogeneity between studies in probiotic research for cancer treatment is evident in various aspects. For instance, different studies investigate the effects of diverse probiotic strains, such as Lactobacillus rhamnosus GG or Lactiplantibacillus plantarum, making it challenging to determine the most effective strains for specific cancer types. Additionally, variations in probiotic doses administered further contribute to heterogeneity, with some studies using higher doses while others opt for lower doses. The duration of probiotic interventions also varies, ranging from a few weeks to several months. Moreover, the heterogeneity extends to the patient populations involved, encompassing different cancer types, stages, and treatments. Lastly, variations in study designs, including sample sizes, control groups, and outcome measures, further hinder comparability and interpretation of results. Addressing these heterogeneities through standardized protocols, larger sample sizes, and consistent methodologies will enhance the reliability and generalizability of conclusions drawn from probiotic research in cancer treatment.

The optimal delivery methods, formulations, or combination strategies of probiotics to maximize efficacy are still being investigated [74]. Different strains of probiotics have different effects on the gut microbiota and the immune system. It is important to choose strains that have been shown to be beneficial for the specific condition being treated. Probiotics can be delivered in a variety of formulations with different benefits [75]. Oral delivery is the most common method of probiotic administration. However, other methods, such as rectal delivery and vaginal delivery, are also being investigated. Enteric-coated granules and freeze drying are methods being proposed recently [76]. The optimal dosage of probiotics is still being investigated [77]. Probiotics may be more effective when combined with other therapies [78][79][80].

## Conclusion

In conclusion, while probiotics have shown promise in modulating the gut microbiota, immune system, and inflammatory responses, their specific role in hematological cancer patients with compromised immunity remains uncertain. Further research is imperative to establish the efficacy and safety of probiotics in this population, including the identification of optimal strains, doses, and treatment durations. By addressing these knowledge gaps, we can pave the way for evidence-based and personalized approaches that optimize the use of probiotics in the comprehensive management of hematological cancers.

## Conflict of Interest

None.
